# Role for ovarian hormones in purinoceptor-dependent natriuresis

**DOI:** 10.1186/s13293-020-00329-0

**Published:** 2020-09-14

**Authors:** Eman Y. Gohar, Malgorzata Kasztan, Shali Zhang, Edward W. Inscho, David M. Pollock

**Affiliations:** Division of Nephrology, Department of Medicine, University of Alabama at Birmingham, 720 20th St S, Kaul 840, Birmingham, AL 35233 USA

**Keywords:** Purinoceptors, Renal medulla, Sodium excretion, Ovariectomy

## Abstract

**Background:**

Premenopausal women have a lower risk of hypertension compared to age-matched men and postmenopausal women. P2Y_2_ and P2Y_4_ purinoceptor can be considered potential contributors to hypertension due to their emerging roles in regulating renal tubular Na^+^ transport. Activation of these receptors inhibits epithelial Na^+^ channel activity (ENaC) via a phospholipase C (PLC)-dependent pathway resulting in natriuresis. We recently reported that activation of P2Y_2_ and P2Y_4_ receptors in the renal medulla by UTP promotes natriuresis in male and ovariectomized (OVX) rats, but not in ovary-intact females. This led us to hypothesize that ovary-intact females have greater basal renal medullary activity of P2 (P2Y_2_ and P2Y_4_) receptors regulating Na^+^ excretion compared to male and OVX rats.

**Methods:**

To test our hypothesis, we determined (i) the effect of inhibiting medullary P2 receptors by suramin (750 μg/kg/min) on urinary Na^+^ excretion in anesthetized male, ovary-intact female, and OVX Sprague Dawley rats, (ii) mRNA expression and protein abundance of P2Y_2_ and P2Y_4_ receptors, and (iii) mRNA expression of their downstream effectors (PLC-1δ and ENaCα) in renal inner medullary tissues obtained from these three groups. We also subjected cultured mouse inner medullary collecting duct cells (segment 3, mIMCD3) to different concentrations of 17ß-estradiol (E_2_, 0, 10, 100, and 1000 nM) to test whether E_2_ increases mRNA expression of P2Y_2_ and P2Y_4_ receptors.

**Results:**

Acute P2 inhibition attenuated urinary Na^+^ excretion in ovary-intact females, but not in male or OVX rats. We found that P2Y_2_ and P2Y_4_ mRNA expression was higher in the inner medulla from females compared to males or OVX. Inner medullary lysates showed that ovary-intact females have higher P2Y_2_ receptor protein abundance, compared to males; however, OVX did not eliminate this sex difference. We also found that E_2_ dose-dependently upregulated P2Y_2_ and P2Y_4_ mRNA expression in mIMCD3.

**Conclusion:**

These data suggest that ovary-intact females have enhanced P2Y_2_ and P2Y_4_-dependent regulation of Na^+^ handling in the renal medulla, compared to male and OVX rats. We speculate that the P2 pathway contributes to facilitated renal Na^+^ handling in premenopausal females.

## Introduction

Women are largely protected from hypertension during their premenopausal age, compared to age-matched men [1]. The risk of hypertension is increased after menopause which is a state characterized by the cessation of ovarian production of the female sex steroid, estradiol (E_2_) [2, 3]. Data suggest that E_2_ exerts protective effects on cardiovascular and renal health in premenopausal females [4–7].

Maintenance of Na^+^ balance and efficient renal Na^+^ excretion is a fundamental aspect in the regulation of blood pressure [8, 9]. Multiple overlapping systems contribute to the regulation of renal tubular reabsorption of Na^+^. Data implicate an important role for purinoceptors (P2 receptors) in controlling urinary Na^+^ excretion [10, 11]. P2 receptors are classified into ligand-gated ion channels, P2X_1–7_, and G protein-coupled receptors, P2Y_1,2,4,6,11–14_ receptors [11–13]. Recently, it has been shown that P2Y_2_ and P2Y_4_ purinoceptor subtypes play a central role in promoting urinary Na^+^ excretion and influencing blood pressure [10, 11]. Genetic deletion of P2Y_2_ receptors results in elevated renal Na^+^ reabsorption and hypertension [14]. Increased dietary salt increases ATP release from collecting duct cells. Activation of P2Y_2_ and P2Y_4_ purinoceptors by ATP increases intracellular Ca^2+^ and reduces epithelial Na^+^ channel (ENaC) activity in collecting ducts via phospholipase C (PLC), resulting in a natriuretic effect [10, 11, 15, 16].

Regulation of Na^+^ excretion via the P2-mediated signaling cascade has been studied almost exclusively in males. Despite ample evidence for sex differences in P2-mediated signaling outside the kidney [17–19], sex differences in the renal P2 system are poorly understood. We recently reported that activation of renal medullary P2Y_2_ and P2Y_4_ receptors by UTP infusion for 1 h promotes natriuresis in male rats [20]. In addition, we showed that 1 h of UTP infusion to the renal medulla did not stimulate natriuresis in ovary-intact female rats while ovariectomy unmasked UTP-induced natriuretic actions [21], pointing to sex-related differences in P2-mediated inhibitory tone on tubular Na^+^ reabsorption.

The goal of the current study was to test whether renal medullary P2 (P2Y_2_ and P2Y_4_) receptors exert a greater role in regulating Na^+^ excretion in ovary-intact female rats, compared to males, and whether ovariectomy blunts P2-dependent natriuretic pathway. Specifically, we hypothesize that ovary-intact female rats have activated renal medullary P2Y_2_ and P2Y_4_ receptors, which leads to PLC activation, ENaC inhibition, and consequently enhanced Na^+^ excretion, compared to male and ovariectomized (OVX) rats. We determined the expression of P2Y_2_ and P2Y_4_ receptors and their downstream effectors in the renal inner medulla. Of note, the inner medullary collecting duct plays an integral role in fine-tuning Na^+^ reabsorption [22]. Given that the female sex steroid, E_2_, has an established role in mediating sex differences in cardiovascular and renal disease females [4–7], we also investigated the impact of E_2_ treatment of mouse inner medullary collecting duct segment 3 (mIMCD3) cells on P2Y_2_ and P2Y_4_ receptor mRNA expression.

## Methods

### Animals

Male and female (16-20 weeks of age) Sprague Dawley (SD) rats from Envigo (Indianapolis, IN) were used. All animal protocols were in accordance with the ARRIVE guidelines [23] and the Guide for the Care and Use of Laboratory Animals and were approved by the University of Alabama at Birmingham Institutional Animal Care and Use Committee. Animals were housed in a temperature (18-23 °C) controlled room with a 12:12-h light-dark cycle with free access to food and water. Animals were maintained on 7917 irradiated NIH-31 mouse/rat diet (0.8% NaCl, Envigo).

### Ovariectomy

Rats (13-17 weeks of age) were subjected to bilateral ovariectomy, as detailed in our previous studies [24]. Three weeks later, acute intramedullary infusion experiments were performed. Briefly, female rats were anesthetized using isoflurane (2%, 502017, Vetone). Bilateral incisions were made on both sides of the back. Ovaries were then exteriorized, tied off and removed. Then, the muscle layer was sewed and the incision was closed using wound clips.

### Acute intramedullary infusion

**Fig. 1:**
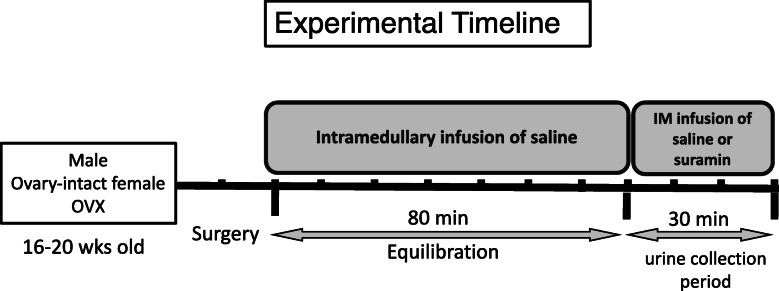
Schematic presentation of the experimental timeline employed in intramedullary infusion experiments

Male, ovary-intact female, and OVX rats were anesthetized using thiobutabarbitone (Inactin, hydrate, 100 mg/kg, ip, T133, Sigma-Aldrich Co.) and surgically prepared similar to our previous studies [20]. Briefly, animals were maintained on a heated surgical table to maintain body temperature at 37 °C. Tracheotomy was performed using PE-205 to facilitate breathing. The femoral vein was catheterized (PE-50) to allow fluid resuscitation with 3% bovine serum albumin in phosphate-buffered saline at a rate of 1.2 ml/h to maintain euvolemia. The femoral artery was catheterized (PE-50) to measure mean arterial pressure (MAP). A 5-6 mm catheter (PE-10) was inserted into the renal medulla of the left kidney to deliver fluids to the renal medullary interstitium at a rate of 0.5 ml/h. Positioning of the catheter tip was confirmed by kidney dissection at the end of each experiment. Urine was collected from the infused kidney by ureter catheterization (PE-10). Animals were allowed to equilibrate for 80 min during which saline was infused into the renal medulla. This was followed by intramedullary infusion of the P2 antagonist, suramin (750 μg/kg/min [20], S2671, Sigma-Aldrich Co., dissolved in saline) or vehicle for a 30 min urine collection period (Fig. 1). Urinary electrolyte levels were measured using an atomic absorption spectrometer (iCE 3000 series paired with a CETAC ASX-520 AutoSampler, ThermoFisher Scientific) in the flame photometry mode.

### Gene expression assessment by RT-PCR

RNA was isolated from tissues or cultured cells using a Purelink Mini extraction kit (12183018A, ThermoFisher Scientific) or a Purelink miRNA extraction kit (K157001, ThermoFisher Scientific), respectively, according to manufacturer’s instructions. The isolated RNA was reverse transcribed using a QuantiTect Reverse Transcription kit (205311, Qiagen). mRNA was quantified by RT-PCR (CFX96 Real-Time System, BIORAD) using TaqMan primer gene expression assays with rat P2Y_2_ receptor (Rn02070661_s1), rat P2Y_4_ (Rn02133903_s1), rat β-Actin (Rn00667869_m1), mouse P2Y_2_ receptor (Mm02619978_s1), mouse P2Y_4_ receptor (Mm00445136_s1) and mouse β-Actin (Mm02619580_g1) primers. mRNA expression was quantified relative to β-Actin using 2^−ΔΔCt^ method. Gene expression data are expressed as the fold change from the mean mRNA expression values in ovary-intact female rats.

### Western blotting

Renal inner medullary tissues were processed as previously described [25, 26]. Briefly, inner medullae were homogenized in radioimmunoprecipitation assay lysis buffer (RIPA, 9806, Cell Signaling Technology) with a protease inhibitor cocktail (One complete tablet in 10 ml lysis buffer; 11697498001, Roche Diagnostics) using Bullet Blender Tissue Homogenizer (Next Advance Inc.). The protein concentration was measured by a Bradford assay (Bio-Rad Laboratories). Inner medulla protein lysates were transferred and incubated with rabbit anti-P2Y_2_ or anti-P2Y_4_ receptor primary antibody (APR-010, APR-006, respectively, Alomone Labs) at 1:1500 dilution at 4 °C overnight. The blots were then incubated for 1 h at room temperature with anti-rabbit IgG, HRP-linked secondary antibody (7074, Cell Signaling Technology) at 1:7000 dilution. Images were developed after exposure to X-ray film. The blots were then re-probed with anti-β-actin (A2228, Sigma-Aldrich Co.) at 1:10000 dilution as a loading control. Relative band densities were quantified using AlphaEaseFC™ software version 3.1.2 (Genetic Technologies Inc.). Densitometry results are expressed as the fold change from the mean values in ovary-intact female rats.

### Cell culture

mIMCD-3 cells (ATYCC CRL-2123, American Type Culture Collection) were cultured as previously described [27] in Dulbecco’s Modified Eagle Medium (F12, ThermoFisher Scientific) containing 10% fetal bovine serum (ThermoFisher Scientific) and 1% penicillin-streptomycin (ThermoFisher Scientific). Cells were incubated at 37 °C in 5% CO_2_-95% air. P*assages 4–6* were used. Cells were grown in 12-well plates and allowed to reach 100% confluency. Cells were serum starved for 3 h, then they were treated with 17ß-estradiol (E_2_, E2758, Sigma-Aldrich Co.) or vehicle for 24 h at final concentrations of 10, 100, or 1000 nM. E_2_ was dissolved in 0.1% ethanol (molecular grade, E7023, Sigma-Aldrich Co.). Values reported are means ± SE and represent results of cells from three experiments with cell lysates assayed in triplicate.

### Statistical analysis

**Fig. 2:**
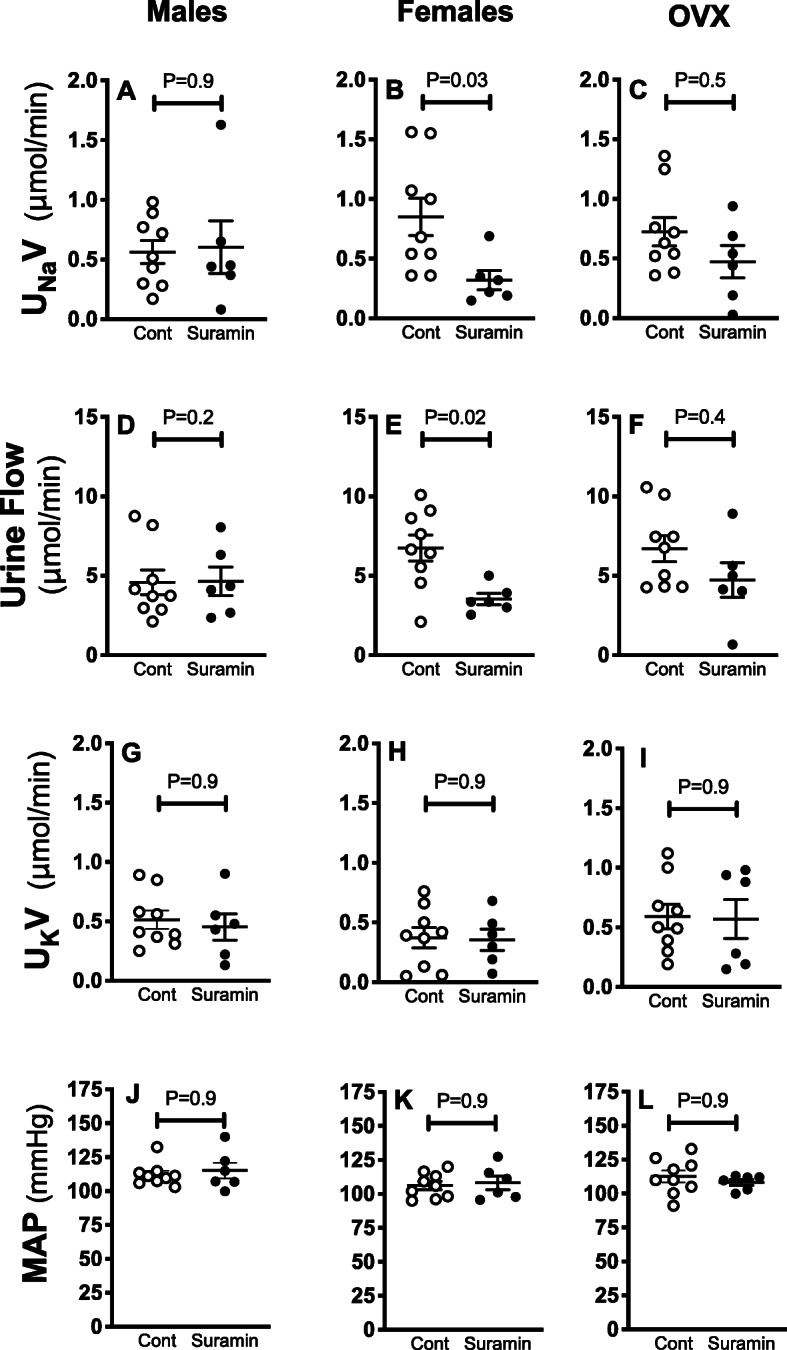
Anti-natriuretic response to renal medullary P2 blockade in ovary-intact female rats only. Urinary Na^+^ excretion (U_Na_V) (**a**-**c**), urine flow (UV) (**d**-**f**), urinary K^+^ excretion (U_K_V) (**g**-**i**), and mean arterial blood pressure (MAP) (**j-l**) in anesthetized male, ovary-intact female, and OVX Sprague Dawley rats receiving renal medullary interstitial infusions of suramin (P2 antagonist, 750 μg/kg/min) or vehicle. *n* = 6-9 in each group. Statistical comparisons performed by two-way ANOVA followed by Sidak’s post hoc tests. ANOVA results: U_Na_V: *P*_interaction_ = 0.1, *P*_suramin_ = 0.04, *P*_sex_ = 0.9, UV: *P*_interaction_ = 0.2, *P*_suramin_ = 0.02, *P*_sex_ = 0.4; U_K_V: *P*_interaction_ = 0.9, *P*_suramin_ = 0.7, *P*_sex_ = 0.2; MAP: *P*_interaction_ = 0.8, *P*_suramin_ = 0.7, *P*_sex_ = 0.2

Statistical tests used for each data set are specified in each figure legend. This includes analysis by one-way ANOVA followed by assessment of differences between the means of the groups using Sidak’s multiple comparison tests. In cell culture studies, Dunnett’s post hoc test was used to compare every mean with a single control mean [28, 29] (Fig. 5). Two-way ANOVA followed by assessment of differences between the means of the groups using Sidak’s post hoc tests was used for analysis of Fig. 2 data (data are presented in different panels for clarity). Data are presented as means ± SEM, with a probability of *p* < 0.05 considered significant. Statistical analysis was performed using GraphPad Prism version 8.

## Results

### Natriuretic role for P2 receptors in the renal medulla

To determine the contribution of renal medullary P2 receptors, we infused the P2 antagonist, suramin, into the renal medulla of male, ovary-intact female, and OVX rats (Fig. 2). Suramin significantly decreased urinary Na^+^ excretion and urine flow relative to vehicle-infused values (Fig. 2b, e) only in ovary-intact females. Urine flow and Na^+^ excretion did not significantly change during medullary blockade of P2 receptors in male or OVX rats (Fig. 2a, c, d, f). Urinary K^+^ excretion (Fig. 2g-i) and MAP (Fig. 2j-l) were not significantly altered by suramin in males, ovary-intact females, or OVX.

### Renal inner medullary P2Y_2_ and P2Y_4_ receptor mRNA expression

**Fig. 3:**
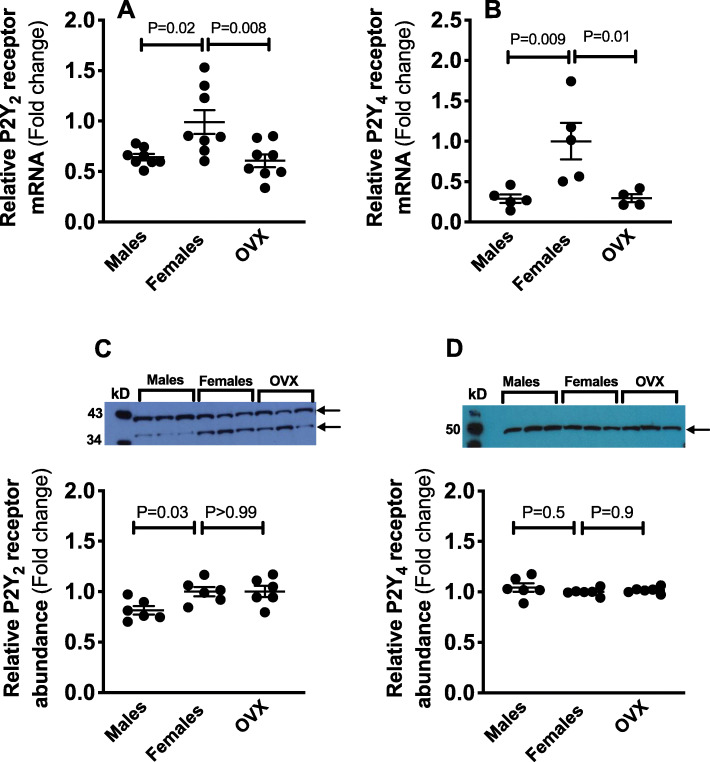
Inner medullary P2Y_2_ and P2Y_4_ receptor mRNA expression and protein abundance. Relative mRNA expression and total protein abundance of P2Y_2_ (**a**, **c**) and P2Y_4_ (**b**, **d**) receptor in the inner medulla from male, ovary-intact female, and OVX Sprague Dawley rats (representative Western blots are presented). Gene expression and protein abundance values represent fold change from ovary-intact female levels. *n* = 4-8 rats in each group. Statistical comparisons performed using one-way ANOVA followed by Sidak’s post hoc tests. ANOVA results: P2Y_2_ mRNA: *P* = 0.004, *F* = 7.1; P2Y_4_ mRNA: *P* = 0.007, *F* = 8.0; P2Y_2_ protein: *P* = 0.02, *F* = 4.9; P2Y_4_ protein: *P* = 0.5, *F* = 0.7

We determined P2Y_2_ and P2Y_4_ receptor expression in inner medullary tissues from kidneys obtained from male, ovary-intact female, and OVX rats. We found that P2Y_2_ receptor mRNA expression is higher in the inner medulla of ovary-intact female rats compared to males (Fig. 3a). This sex difference was eliminated by ovariectomy (Fig. 3a). Renal inner medullary P2Y_4_ receptor mRNA expression followed the same pattern as P2Y_2_ receptor mRNA expression (Fig. 3b).

### Renal inner medullary P2Y_2_ and P2Y_4_ receptor protein abundance

As expected, based on the molecular weight of P2Y_2_ receptor, Western blots demonstrated an intense band at approximately 42-kD (Fig. 3c), which was completely ablated by preincubation with the blocking peptide (Supplemental Fig. 1A). No differences were evident in the intensity of this band between groups. Western blots for P2Y_2_ receptor consistently demonstrated another slightly lower molecular weight band (approximately 36-kD), which also underwent complete ablation when incubated with the blocking peptide (Supplemental Fig. 1A). This band may represent a posttranslational modified form of the P2Y_2_ receptor, but this will require further investigation. This 36-kD band was significantly higher in ovary-intact females, in comparison with males (*p* = 0.03); however, ovariectomy did not impact this 36-kD band. Overall, the combined mean densities of the two bands for P2Y_2_ receptor were higher in inner medulla from kidneys obtained from ovary-intact females, compared to males, consistent with the mRNA data (Fig. 3c). Ovariectomy did not change the combined mean densities of the two bands for P2Y_2_ receptor (Fig. 3c).

Western blots of renal inner medullary lysates for P2Y_4_ receptor showed a band (approximately 50-kD, Fig. 3d), which may represent the glycosylated form of P2Y_4_ receptor [30]. Importantly, preincubation with P2Y_4_ blocking peptide abladed this band (Supplemental Fig. 1B). As quantified in Fig. 3d, the relative intensity of this band was not different between male, ovary intact, or OVX female rats.

### Renal inner medullary PLC-1δ and ENaCα mRNA expression

**Fig. 4:**
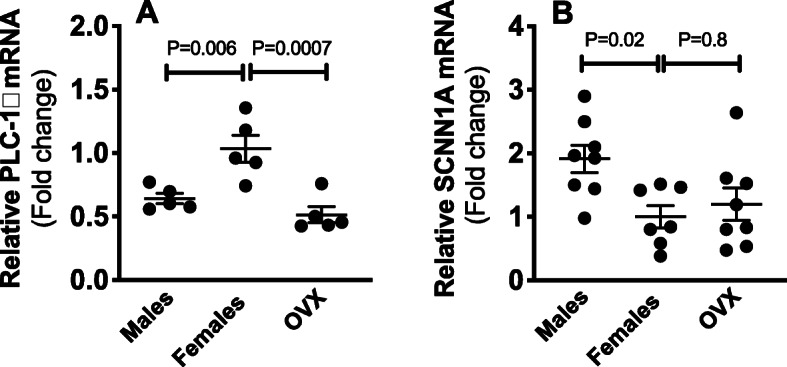
Inner medullary PLC-1δ and SCNN1A mRNA expression. Relative mRNA expression of PLC-1δ (**a**) and SCNN1A (ENaCα) (**b**) in the inner medulla from male, ovary-intact female, and OVX Sprague Dawley rats. Gene expression values represent fold change from ovary-intact female levels. *n* = 5-8 rats in each group. Statistical comparisons performed using one-way ANOVA followed by Sidak’s post hoc tests. ANOVA results: PLC-1δ: *P* = 0.001, *F* = 13.0; SCNN1A: *P* = 0.02, *F* = 4.7

Downstream of purinoceptor activation, PLC-1δ-dependent inhibition of ENaC activity was shown to promote natriuresis [10, 11, 15, 16]. We determined the mRNA expression levels of PLC-1δ and ENaCα (SCNN1A) in kidneys from male, ovary-intact female, and OVX rats. We found that the mRNA expression of PLC-1δ was higher in the renal inner medulla of ovary-intact female rats compared to males (Fig. 4a). Ovariectomy abolished this male-female difference in PLC-1δ mRNA expression (Fig. 4a). We demonstrated that inner medullary ENaCα mRNA expression was markedly lower in ovary-intact female rats compared to males (Fig. 4b). In contrast, no differences were detected in the mRNA expression of inner medullary ENaCα in response to ovariectomy (Fig. 4b).

### E_2_ increases P2Y_2_ and P2Y_4_ receptor mRNA

**Fig. 5:**
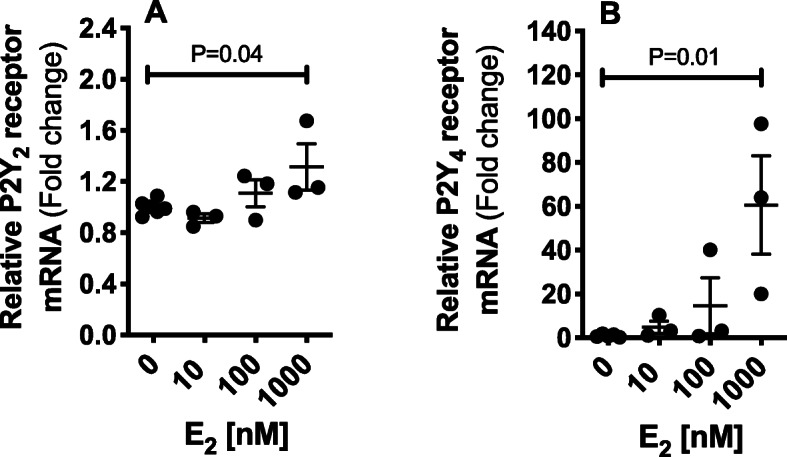
E_2_ promotes P2Y_2_ and P2Y_4_ receptor mRNA expression in the inner medullary collecting duct cells (IMCD-3). P2Y_2_ (**a**) and P2Y_4_ (**b**) receptor mRNA expression in IMCD-3 cells incubated with E_2_ (1, 10, 100, 1000 nM) or vehicle for 24 h. *n* = 3-6 in each group. Statistical comparisons performed using one-way ANOVA followed by Dunnett’s post hoc tests. ANOVA results: P2Y_2_ mRNA: *P* = 0.04, *F* = 3.7; P2Y_4_ mRNA: *P* = 0.01, *F* = 6.1

To identify the impact of the female sex steroid, E_2_, on P2Y_2_ and P2Y_4_ receptor mRNA expression in the inner medullary collecting ducts, we treated mIMCD3 cells with different doses of E_2_ (10, 100, 1000 nM) or vehicle (0.1% ethanol) for 24 h. We observed that E_2_ dose dependently increases the mRNA expression of P2Y_2_ and P2Y_4_ receptors in mIMCD3 cells (Fig. 5).

## Discussion

The current report establishes an important role for sex and sex steroids in regulating P2-mediated Na^+^ excretion. Our results showed that (i) infusion of the P2 antagonist, suramin, to the renal medulla attenuated urinary Na^+^ excretion in ovary-intact female rats, but not in male or OVX rats, (ii) OVX abolished the male-female difference in the mRNA expression of P2Y_2_, P2Y_4_ receptors and PLC-1δ in the inner medulla of the kidney, and (iii) the protein abundance of the P2Y_2_ receptor is higher in renal inner medulla from ovary-intact female rats, compared to males. (iv) We also provide in vitro evidence that E_2_ upregulates the mRNA expression of P2Y_2_ and P2Y_4_ receptors in mIMCD3. All together, these findings suggest an interaction between E_2_ and P2 signaling in the inner medulla to promote renal Na^+^ excretory function under basal physiological conditions in ovary-intact females.

Studies suggest that females have a more advanced capacity to excrete salt, compared to age-matched males [31, 32]. This female advantage appears in both experimental animals and humans [31–33]. Renal Na^+^ handling is a complex and highly regulated physiological process that involves multiple mechanistic pathways. The renal tubular P2 signaling plays important roles in regulating urinary Na^+^ excretion [10, 11], however, whether P2-dependent renal signaling is differentially regulated based on sex and sex hormones are not clear. We recently reported that infusion of the P2Y_2_ and P2Y_4_ agonist, UTP [34], into the renal medulla evokes natriuresis in male and OVX rats, but not ovary-intact females [20, 21]. This observation directed us to focus on the P2Y_2_/P2Y_4_ signaling cascade as a potential contributor to sex-related physiological differences in renal salt handling. This is particularly relevant to evidence demonstrating that sex hormones regulate extrarenal purinoceptor signaling [35–38].

To elucidate the role of endogenous activation of P2 receptors on urinary Na^+^ excretion under basal physiological conditions, we determined the effect of intramedullary infusion of the non-selective P2 antagonist, suramin, on basal urinary Na^+^ excretion in male rats and female rats with and without ovaries. We found that suramin attenuates urinary Na^+^ excretion in ovary-intact females, but not in males or OVX rats (Fig. 5), indicating that endogenous activation of P2 receptors inhibits tubular Na^+^ reabsorption in ovary-intact females, but not males or OVX females. The impact of antagonism of P2 receptors on renal P2Y_2_ and P2Y_4_ expression level and localization is not clear. Chronic treatment with suramin for 3 months reduces myocardial P2Y_2_ receptor abundance in a mouse model of muscular dystrophy [39]. We presume that short-term infusion of suramin for 30 min does not impact P2Y_2_ and P2Y_4_ expression; however, it is possible that acute effects of suramin may involve changes in the localization/activation of P2Y_2_ and/or P2Y_4_ receptors.

Given that suramin is a non-selective blocker for P2 receptors, our results do not provide us with definite clues regarding which P2 receptor subtype(s) enhance(s) urinary Na^+^ excretion in ovary-intact females. Evidence primarily points to P2Y_2_ and P2Y_4_ as important players in evoking natriuresis and regulating blood pressure [10, 11]. Thus, in the present study, we focused on studying aspects of the P2Y_2_ and P2Y_4_-mediated signaling cascades as potential natriuretic pathways that may contribute to sex-related differences in Na^+^ excretion. Additional studies are needed to fully understand the impact of sex and sex steroids on the control of Na^+^ excretory function by P2Y and P2X receptors.

The signaling mechanisms by which P2 receptor activation evokes natriuresis involves inhibition of ENaC activity via a PLC-dependent pathway [10, 11, 15, 16]. It has been shown that inhibiting P2 receptors rapidly enhances ENaC activity [16]. Experimental evidence documents the expression and functionality of P2Y_2_ and P2Y_4_ receptors in the collecting duct [11, 13, 16, 40]. P2Y_2_ knockout mice are hypertensive, possibly due to ENaC hyperactivity leading to enhanced renal Na^+^ reabsorption [14, 16]. The current study demonstrates that the renal inner medulla from ovary-intact female rats have higher mRNA expression of P2Y_2_ and P2Y_4_ receptors and PLC, compared to males. Importantly, this male-female difference is eliminated by ovariectomy. Consist with increased activity of P2 receptors in females, we found that ENaC mRNA expression was markedly lower within the inner medulla from female rats compared to males. However, we did not observe OVX-related differences in inner medullary ENaCα mRNA expression. It is possible that there are sex and sex hormonal-related differences in the activity, rather than the expression, of this ion channel. It has been demonstrated that the distal nephrons of female rats have a higher abundance of cleaved forms of ENaCα and γ, compared to males [31]. Given that PLC signaling couples P2 receptor to ENaC [16], sex-dependent regulation of PLC may present an indirect regulatory role for sex on ENaC activity. Further functional studies are necessary to identify sex and sex hormonal-dependent modulation of ENaC activity.

Similar to our mRNA data, the protein abundance of P2Y_2_ receptor was higher in ovary-intact females, compared to males. This sex difference at the protein level was not abolished by OVX. Despite our observation that ovary-intact female rats exhibit higher P2Y_4_ receptor mRNA expression in their renal inner medulla compared to males or OVX rats, no differences were observed at the total protein level. The disconnect between the level of mRNA expression and the receptor protein levels for P2Y_4_ receptor may reflect differences in programmed receptor destruction or post-translational modifications, rather than differences in transcription. Further studies are needed to address potential sex and sex hormone-dependent differences in the processing of mRNA to translation, modification, localization, and protein degradation.

E_2_ is pivotal for the maintenance of cardiovascular and renal health in females [4, 5]. We provide in vitro evidence that E_2_ dose-dependently increases mRNA expression of P2Y_2_ and P2Y_4_ receptors in mIMCD3 cells. These data are consistent with our finding that ovary-intact female rats have an enhanced renal P2Y_2_/P2Y_4_ signaling system, compared to males. In contrast, ovariectomy did not alter the protein abundance of P2Y_2_ and P2Y_4_ receptors. Thus, we propose that other sex-specific factors, besides E_2_, regulate the renal medullary P2 system. Notably, the gene coding for the P2Y_4_ receptor is located on chromosome X [41]. Whether the chromosomal complement plays a role in regulating the renal P2Y_2_/P2Y_4_ signaling system remains to be determined. Importantly, renal estrogen receptor, ER, expression has been shown in multiple studies [42–44]. Binding studies using radiolabeled E_2_ revealed that radioactivity localizes to the proximal tubule and the inner medullary collecting duct [45], which is relevant to our findings in mIMCD3 cells. Data showed that classical ER, ERα, and ERβ, and membrane-associated ER, G protein-coupled ER, are expressed in the collecting ducts [46]. However, the exact relationship between the ER and P2 signaling systems in the kidney is not clear.

Sex-specific discrepancies in the expression of renal ER have been previously reported [43]. Hutson et al. demonstrated that GPER mRNA expression was higher in kidneys from female Sprague Dawley rats, compared to males [43]. Consistently, we recently showed that the mRNA expression and protein abundance of GPER within the renal inner medulla from female Sprague Dawley rats is higher than males [47], whereas renal ERα mRNA expression is greater in male, compared to female Sprague Dawley rats [43]. Of note, renal ERα mRNA expression was markedly diminished after OVX in Wistar rats [48]. Other investigators within our group have verified that mIMCD3 cells were originally derived from male mice. Whether the expression of ER in IMCD cells is sexually dimorphic remains to be determined. Given that renal ER expression appears to be regulated by sex and sex hormones [43, 47, 48], we speculate that the sex of cells may impact the effect of E_2_ treatment on P2 receptor expression. Future studies are needed to determine the effect of E_2_ on P2Y_2_ and P2Y_4_ in freshly isolated IMCD from male, ovary-intact female, and OVX rats.

Overall, studies in recent years generally reinforce the importance of ovarian hormones in determining the quality of life and prognosis of cardiovascular and renal diseases in female patients. When results of needed studies of the modulatory role of sex hormones on critically important systems involved in the control of Na^+^ homeostasis and blood pressure are available, developing new clinical practice guidelines will be applicable.

### Study limitations

Despite that it is established that OVX is the standard approach for studying the impact of ovarian hormones on female health in preclinical research [49], it is important to note that there are limitations for OVX as a model for the study of postmenopausal females. Aging is a confounding factor that contributes to postmenopausal physiological changes; however, OVX surgery was conducted in the current study in relatively young animals. In addition, OVX results in an abrupt decline in the plasma concentration of ovarian hormones, which is different from the slow nature of the human menopause transition, which typically spans over few (4-6) years [49]. Due to the sudden nature combined with the age of the animals employed in the current study, OVX accurately models surgical, rather than natural, menopause in women.

In addition to the sex-related differences in the signaling pathway downstream to P2Y_2_ and P2Y_4_ activation in the renal medulla that we identified in the current study, it is possible that there are differences in P2Y_2_ and P2Y_4_ receptor upstream signaling that may contribute to sex differences in P2-mediated natriuresis. Future studies are needed to determine whether there are discrepancies between males and females in renal medullary ATP levels, ecto-ATPases, and Connexin 30 channel expression and function.

### Perspective and significance

To our knowledge, this is the first study showing sex and sex-hormonal related differences in P2-dependent regulation of urinary Na^+^ excretion. This finding may contribute to the lower prevalence of hypertension and enhanced ability to handle salt challenges evident in premenopausal females. Additional studies are necessary to identify the contribution of ATP/P2Y_2_/P2Y_4_/PLC/ENaC signaling cascade to salt sensitivity in postmenopausal female population.

## Supplementary information

**Additional file 1: Supplemental Figure 1.** Determination of the specificity of anti-P2Y_2_ receptor and anti-P2Y_4_ receptor antibodies by immunoblotting. Representative Western blots for inner medullary homogenates from male, ovary-intact female and OVX Sprague Dawley rats incubated with anti-P2Y_2_ receptor antibody (A) or anti-P2Y_4_ receptor antibody (APR-010, APR-006, respectively, Alomone Labs) in the presence (right) and absence (left) of the respective blocking peptide.

## Data Availability

The data that support the findings of this study are available from the corresponding author upon reasonable request.
