# Supplementary material for: Role for ovarian hormones in purinoceptor-dependent natriuresis
Source: Biol Sex Differ. 2020 Sep 14;11:52. doi: 10.1186/s13293-020-00329-0 (PMC7490965; doi:10.1186/s13293-020-00329-0)

Data Supplement

Role for ovarian hormones in purinoceptor-dependent natriuresis

Eman Y. Gohar, Malgorzata Kasztan, Shali Zhang, Edward W. Inscho, David M. Pollock

Division of Nephrology, Department of Medicine, University of Alabama at Birmingham, Birmingham, AL

Supplemental Figure 1. Determination of the specificity of anti-P2Y_2 receptor and anti-P2Y_4 receptor antibodies by immunoblotting. Representative Western blots for inner medullary homogenates from male, ovary-intact female and OVX Sprague Dawley rats incubated with anti-P2Y_2 receptor antibody (A) or anti-P2Y_4 receptor antibody (APR-010, APR-006, respectively, Alomone Labs) in the presence (right) and absence (left) of the respective blocking peptide.

Supplemental Figure 1.

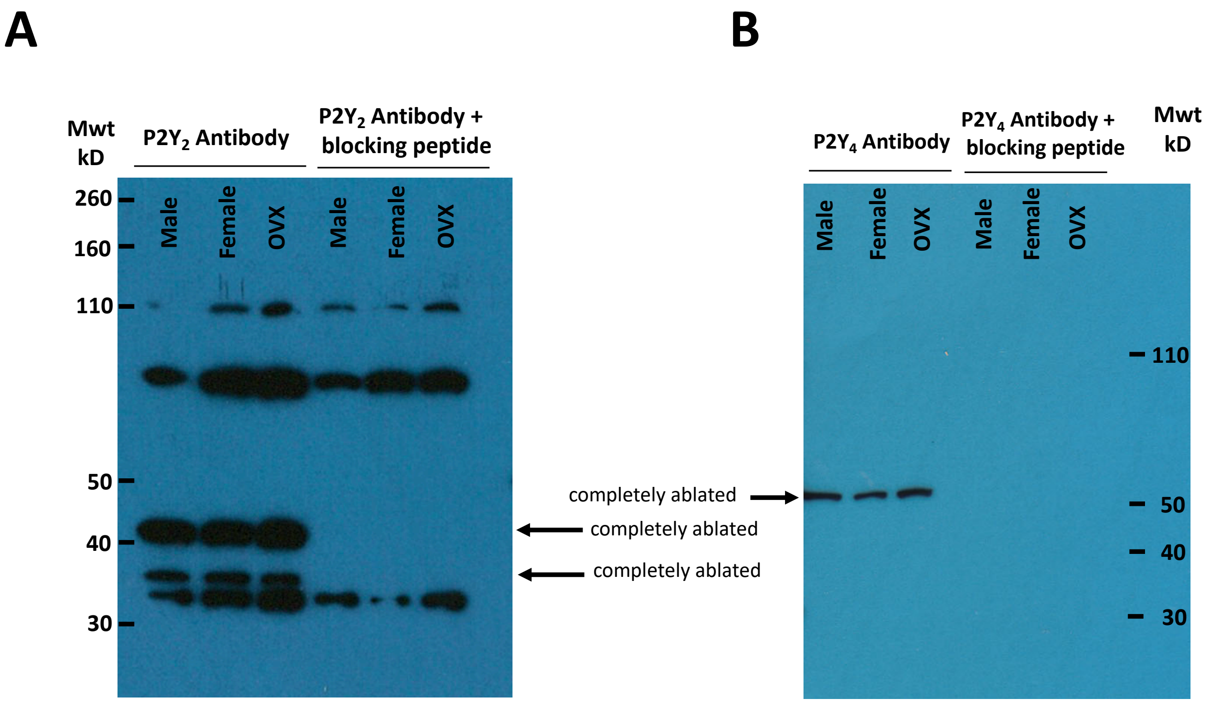

Supplement: Supplementary file 1 — Additional file 1: Supplemental Figure 1. Determination of the specificity of anti-P2Y2 receptor and anti-P2Y4 receptor antibodies by immunoblotting. Representative Western blots for inner medullary homogenates from male, ovary-intact female and OVX Sprague Dawley rats incubated with anti-P2Y2 receptor antibody (A) or anti-P2Y4 receptor antibody (APR-010, APR-006, respectively, Alomone Labs) in the presence (right) and absence (left) of the respective blocking peptide. [file 13293_2020_329_MOESM1_ESM.pdf]
